# Efficient CO_2_ adsorption using chitosan, graphene oxide, and zinc oxide composite

**DOI:** 10.1038/s41598-024-53577-0

**Published:** 2024-02-07

**Authors:** Farnoush Fathalian, Hamidreza Moghadamzadeh, Alireza Hemmati, Ahad Ghaemi

**Affiliations:** 1 Department of Chemical Engineering, Faculty of Engineering, Islamic Azad University, South Tehran Branch, Tehran, Iran; 2 School of Chemical, Petroleum and Gas Engineering, Iran University of Science and Technology, (IUST), Tehran, Iran

**Keywords:** Environmental sciences, Engineering, Materials science

## Abstract

This study was deeply focused on developing a novel CTS/GO/ZnO composite as an efficient adsorbent for CO_2_ adsorption process. To do so, design of experiment (DOE) was done based on RSM-BBD technique and according to the DOE runs, various CTS/GO/ZnO samples were synthesized with different GO loading (in the range of 0 wt% to 20 wt%) and different ZnO nanoparticle’s loading (in the range of 0 wt% to 20 wt%). A volumetric adsorption setup was used to investigate the effect of temperature (in the range of 25–65 °C) and pressure (in the range of 1–9 bar) on the obtained samples CO_2_ uptake capability. A quadratic model was developed based on the RSM-BBD method to predict the CO_2_ adsorption capacity of the composite sample within design space. In addition, CO_2_ adsorption process optimization was conducted and the optimum values of the GO, ZnO, temperature, and pressure were obtained around 23.8 wt%, 18.2 wt%, 30.1 °C, and 8.6 bar, respectively, with the highest CO_2_ uptake capacity of 470.43 mg/g. Moreover, isotherm and kinetic modeling of the CO_2_ uptake process were conducted and the Freundlich model (R^2^ = 0.99) and fractional order model (R^2^ = 0.99) were obtained as the most appropriate isotherm and kinetic models, respectively. Also, thermodynamic analysis of the adsorption was done and the ∆H°, ∆S°, and ∆G° values were obtained around − 19.121 kJ/mol, − 0.032 kJ/mol K, and − 9.608 kJ/mol, respectively, indicating exothermic, spontaneously, and physically adsorption of the CO_2_ molecules on the CTS/GO/ZnO composite’s surface. Finally, a renewability study was conducted and a minor loss in the CO_2_ adsorption efficiency of about 4.35% was obtained after ten cycles, demonstrating the resulting adsorbent has good performance and robustness for industrial CO_2_ capture purposes.

## Introduction

The widespread usage of fossil fuels has led to a significant increase in the concentration of CO_2_ in the Earth’s atmosphere. Previous research suggests that the rapid progression of global warming is mainly attributable to greenhouse gases, with CO_2_ being the primary contributor, which is mostly generated by human activities^1^. These activities involve the emission of carbon dioxide from various industrial sites, including power plants, metallurgical plants, cement factories, and others^2,3^. The sharp elevation of global warming has already resulted in several negative environmental impacts and poses a severe threat to human survival^4^. Diverse CO_2_ capture techniques are utilized within industrial processes, including solvent-based absorption, solid sorbent-based adsorption^5^, membrane processes, and cryogenic separation processes^6^. Of these techniques, CO_2_ absorption using alkali solution and cryogenic approaches present economic impediments that arise from issues such as solvent loss, corrosion risks, elevated pressure and temperature requirements, and significant energy consumption needed for refrigeration purposes^7^. The adsorption technique is a promising method among the various mentioned techniques due to its excessive selectivity, enhanced uptake cability, and low energy requirements for regeneration^8^. Solid sorbents such as graphene oxide^9,10^, mesoporous silica^11,12^, metal oxides (MOs), metal–organic frameworks (MOFs)^13,14^, and porous organic polymers (POPs)^15,16^ are crucial components of the adsorption process.

Within the category of solid adsorbents, graphene-based materials possess an intermediate level of adsorption strength, predominantly exhibiting the physical nature of CO_2_ adsorption on their surface^17^. Consequently, the introduction of polymers presents a potential approach to modify the surface chemistry of the porous materials and enhance their affinity towards CO_2_ adsorption. Porous polymer’s reinforcement using graphene oxide (GO) results in synergistic advantages stemming from both materials, including exceptional mechanical stability, high electrical conductivity, and a high surface area attributed to graphene oxide, coupled with the specific functionalities provided by diverse polymers^18,19^. Polymers themselves have been extensively investigated for CO_2_ capture and their efficacy in this regard depends on crucial properties of the polymer adsorbent, namely composition, porous morphology, and basic nature^18^. Incorporating heteroatoms including nitrogen (N), sulfur (S), or oxygen (O) into a porous polymer’s skeleton facilitates the selective uptake of CO_2_^18,19^. Consequently, polymers containing heteroatoms have been utilized to fortify the pristine graphene structure, such as polyethyleneimine (PEI)^20^, polythiophene^21^, polypyrrole^22,23^, chitosan^24^, or polyaniline^25^. Metal oxides play a vital role in improving the adsorption of CO_2_ in various applications through several mechanisms. First, chemisorption occurs, as metal oxides exhibit a strong affinity for CO_2_ due to Lewis’s acid–base interactions, creating additional CO_2_ binding sites within adsorbents. This enhances adsorption capacity and overall efficiency in CO_2_ capture^26,27^. Second, metal oxide-modified adsorbents typically have an increased surface area, providing more opportunities for CO_2_ molecules to be captured, leading to improved adsorption performance^27^. Additionally, certain metal oxides act as catalysts, accelerating the kinetics of CO_2_ adsorption by facilitating chemical reactions with the adsorbent, thereby enhancing the overall efficiency of CO_2_ capture and conversion^28^. Furthermore, metal oxides contribute to the stability of adsorbents, reducing the risk of material degradation and prolonging the adsorbent's lifespan, ensuring sustainable CO_2_ capture systems^27^.

The synthesis of composites comprising organic polymers and graphene oxide (GO) known as polymer/GO represents a viable approach for modifying the characteristics of the polymer, thereby enhancing its capacity for gas adsorption^29–31^. The polymer/GO composites typically exhibit increased porosity and dispersion forces, owing to the inclusion of GO, which possesses a high atomic density and abundant surface functional groups^32^. Numerous studies have substantiated that the introduction of GO into polymer matrices has a positive influence on both material stability and gas adsorption capabilities. Politakos et al.^33^ introduced a new approach for synthesizing graphene oxide (GO)/biopolymer composite. Their findings emphasize that the quantity of polymer utilized in the composites influences their textural properties, whereby lower amounts result in higher specific surface area and increased functional group content which results in a maximum range of 3.56–3.85 mmol/g at 1 bar and 25 °C. Politakos et al.^34^ engineered the porous structure of the monoliths, resulting in a diverse portfolio of monoliths with various CO_2_ uptake capabilities. The monolith synthesized at the maximum temperature and with the lowest amount of ascorbic acid as a reducing agent exhibited the highest specific surface area, porosity, and level of functionalization achieving a capacity of 2.1 mmol/g at 25 °C and 1 bar pressure. Hsan et al.^35^ presented a sustainable and environmentally friendly approach for developing chitosan (CTS) grafted GO composite as a solid sorbent for the CO_2_ gas capture process. The obtained composite exhibits an adsorption capacity of approximately 0.257 mmol/g at 1 bar for CO_2_ gas, which is significantly higher than that of pure CTS.

Recent research showed that adding metal oxide nanoparticles to adsorbents considerably increased their ability to adsorb CO_2_ molecules^36–38^. Zhou et al.^39^ investigated the impact of Li doping on the structural properties of a MOF material namely HKUST-1 and explored the CO_2_ uptake capability of the resulting material. The morphological characteristics of HKUST-1 remained intact, and the introduction of Li helped rectify certain defects which led to an optimized CO_2_ uptake capacity of 4.3 mmol/g. Krap et al.^40^ synthesized MFM-300(Ga_2_), a Ga-based MOF, using a solvothermal reaction of Ga(NO_3_)_3_ and H_4_L. Method of Fe-doping improved gas uptake capabilities, especially for CO_2_ adsorption, with a 49% enhancement in CO_2_ uptake (2.86 mmol/g at 273 K at 1 bar). MOF-74(Ni, Co) was synthesized, characterized, modified with Pd-loaded AC, and evaluated for CO_2_ adsorption capacity and CO_2_/N_2_ separation efficiency by Adhikari and Lin^41^ Modified MOFs showed enhanced adsorption capacity of 12.24 and 11.42 mmol/g at 298 K and 32 bar and improved bond distances. Another study by Al-Mamoori et al.^42^ presented novel metal oxide-doped CaO adsorbents with high capture capacity, fast kinetics, and long-term stability. Fe and Ga-doping improved adsorption performance, achieving high adsorption capacities (13.7 and 14.2 mmol/g, respectively) and reversible performance. The materials exhibit about a 5% loss in adsorption capacity after ten cycles and exhibit favorable CO_2_ uptake.

Although, chitosan as an amine-rich biopolymer is appropriate for CO_2_ adsorption purposes but the low surface area of this type of porous polymer leads to a low CO_2_ uptake capability which creates some limitations for large-scale CO_2_ capture applications. As a promising technique to tackle this issue, dispersion of a porous material such as GO or a metal oxide such as ZnO nanoparticles on the surface of the chitosan support causes improving adsorbent surface’s heterogeneity and gaining adsorbent’s surface area leading to increasing the CO_2_ uptake capability of the chitosan support^24^. Therefore, this study was conducted aim of developing a novel chitosan/graphene oxide/zinc oxide composite, denoted as CTS/GO/ZnO composite for CO_2_ adsorption purposes. To do so, different composite samples with various compositions were prepared to investigate the effect of GO and ZnO nanoparticles loading weight percent on the composite’s CO_2_ adsorption capacity. By reviewing the literature, it was concluded that many researchers only considered the physiochemical properties of the CTS/GO samples, while developing a predictive model is vital for industrial process design applications. Therefore, the CO_2_ adsorption process modeling was applied using the RSM-BBD approach aim of provide a predictive model to correlate the dependency of the CTS/GO/ZnO adsorbent’s adsorption capacity to the operational condition such as temperature and pressure and synthesis parameters including GO and ZnO loading weight percent. Moreover, the best composition of the CTS/GO/ZnO composite was determined through optimizing the CO_2_ adsorption process, and the obtained adsorbent was characterized using FTIR, XRD, SEM, and BET analysis. Finally, isotherm modeling, kinetic modeling, and thermodynamic assessment of the adsorption process were done to investigate the CO_2_ adsorption mechanism regarding the interactions between CO_2_ molecules and the adsorbent’s surface.

## Materials and methods

### Chemicals

Graphite bulk powder with high purity (> 99.8), Zinc sulfate Heptahydrate (ZnSO_4_. 7H_2_O) with high purity (> 99.95), pure Sodium Hydroxide (NaOH), Cetyl trimethyl ammonium bromide (CTAB), Acetic Acid (100%), sulfuric acid (98%), phosphoric acid (85%), potassium permanganate 99% (KMnO_4_), Hydrogen peroxide 30% (H_2_O_2_), Hydrochloric acid 37% (HCl), and chitosan powder with Deacylation Degree of 79% were supplied from Merck company. All of the mentioned materials were used for the synthesis of the adsorbent without any further purification. During the synthesis procedure, distilled water and HPLC-grade methanol (purity > 99.9%) were utilized for washing and purification purposes.

### Graphene oxide (GO) synthesis

**Figure 1:**
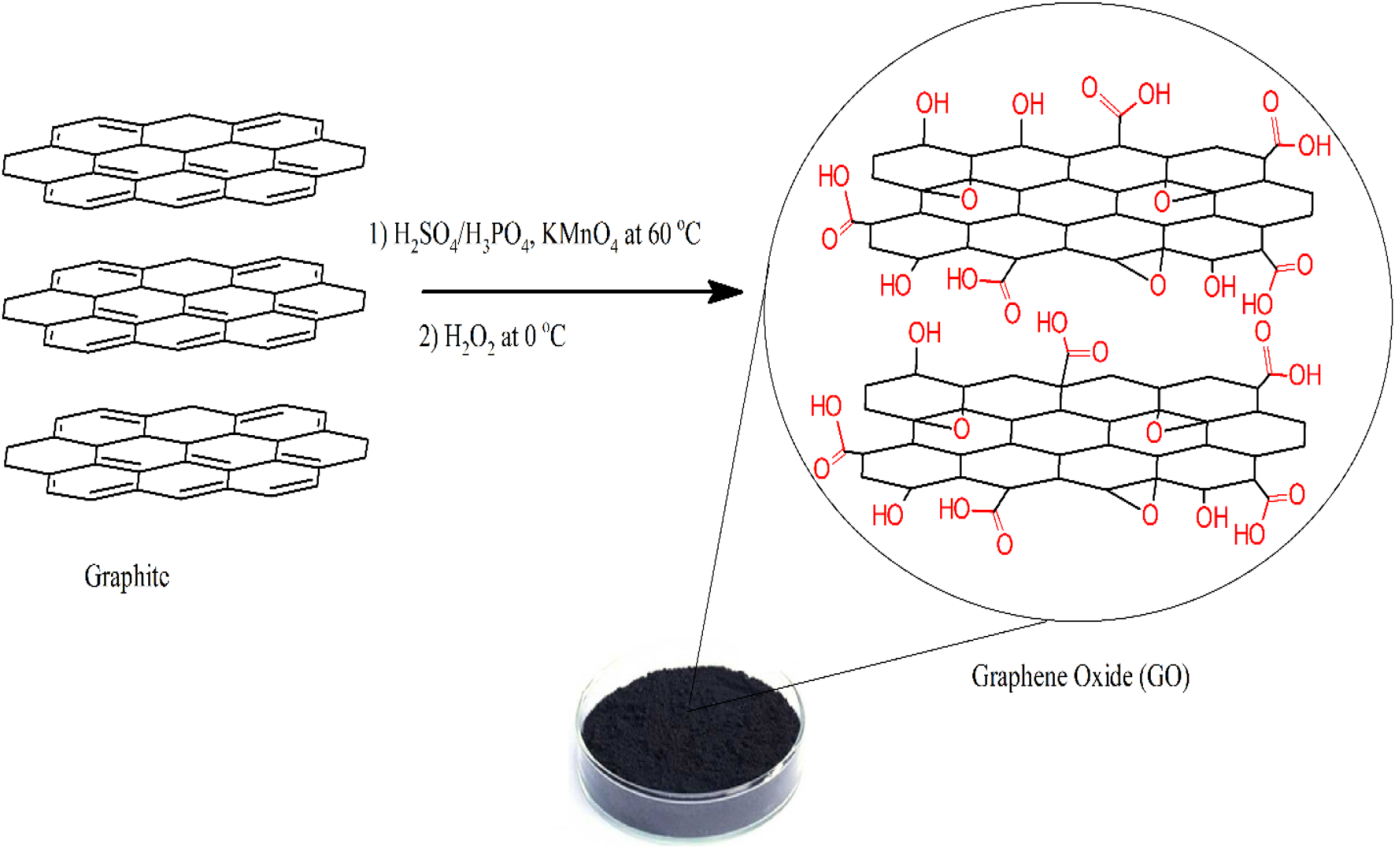
The general procedure of GO Synthesis from graphite powder.

To synthesize the GO nanoparticles from graphite powder, the modified Hummer’s method^43^ was conducted similar to the work conducted by Kumar S.A.K et al.^44^. In a typical procedure, the graphite powder (1.5 g) was charged to a round bottom flask containing the mixture of concentrated H_2_SO_4_/H_3_PO_4_ solution with a ratio of 9:1 (180:20 ml). Next, KMnO_4_ powder (9 g) was introduced to the flask gradually and the mixture temperature was kept at 40 °C, after the complete addition of KMnO_4_ powder the temperature was increased up to 60 °C and the flask contents were mixed for 12 h. Then, the flask contents were cooled down and the mixture was entered into ice (200 ml) containing H_2_O_2_ 30% (2 ml), which resulted in the solution color changing from brown to yellow. Finally, the mixture was filtrated and the obtained solid was purified three times with 250 ml of distilled water, followed by 250 ml of HCL solution (30%) and pure methanol (250 ml). After the purification stage, the GO sample was obtained by centrifugation of the resulting powder at 5000 rpm for 40 min followed by drying inside a vacuum oven at 60 °C for 11 h. The general route for the synthesis of GO from graphite powder is shown in Fig. 1.

### Synthesis of ZnO nanoparticles

Similar to the method reported by Deb A. et al.^45^, the Zinc Oxide (ZnO) nanoparticles were synthesized through precipitation of zinc ions (Zn^2+^) in an alkali solution. In a typical method, the ZnSO_4_.7H_2_O (0.72 g) was dissolved in 120 ml of deionized water (DI) to prepare 120 ml of Zn^2+^ solution (0.025 M). Also, NaOH solution (0.5 M) was prepared by dissolving NaOH (2.0 g) in 120 ml of DI. Next, the NaOH solution was gradually added to the Zn^2+^ solution to precipitate zinc ions, then 20 ml CTAB solution (100 mg/l) was added to the mixture to prevent ZnOH crystal growth. Finally, the obtained solid was filtrated and purified with distilled water and methanol several times and the purified sample was calcined at 420 ^◦^C for 3 h which yielded the ZnO nanoparticles.

### Synthesis of CTS/GO/ZnO composite

**Figure 2:**
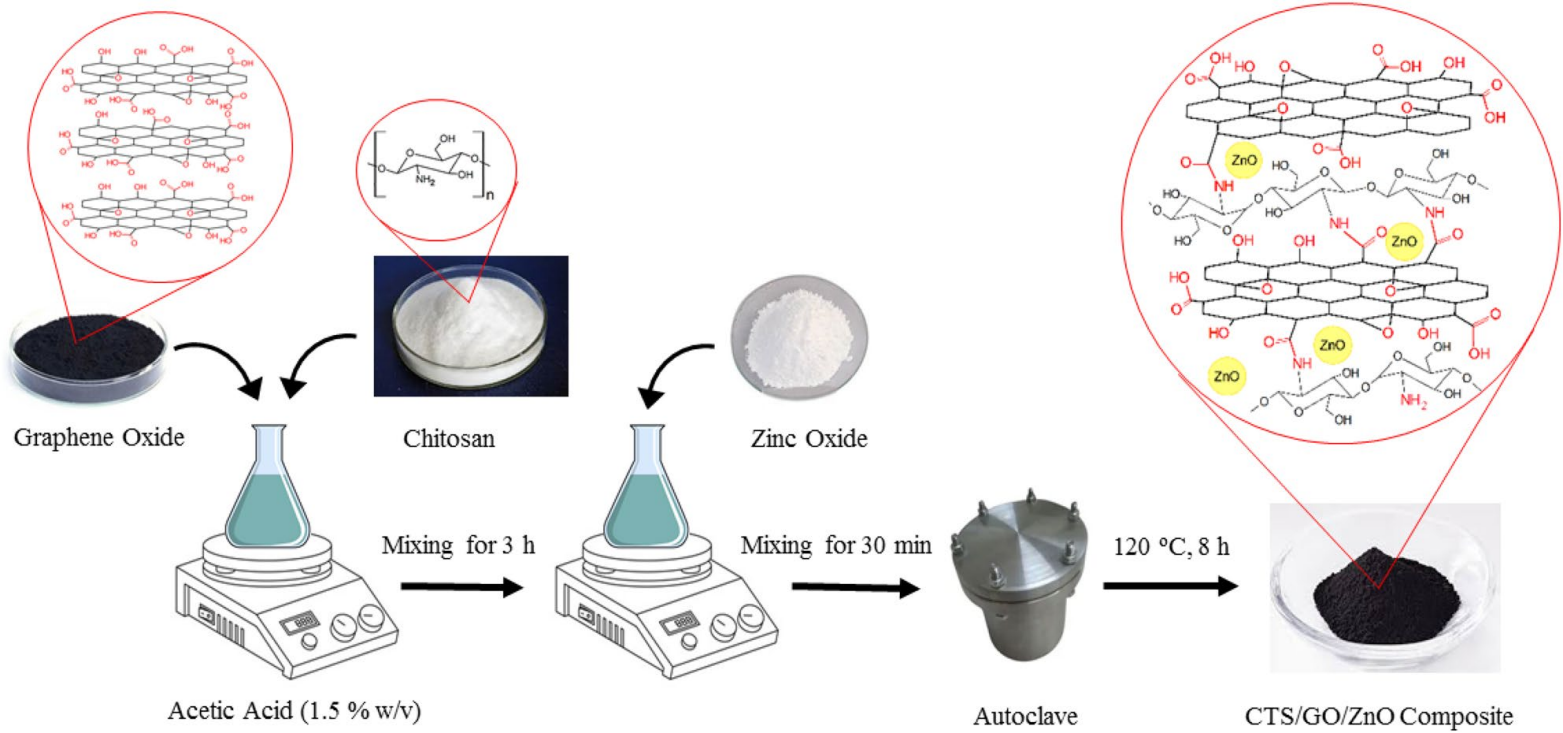
General procedure of synthesis of the CTS/GO/ZnO composite.

The CTS/GO/ZnO composite samples with various weight percent of the GO and ZnO nanoparticles were synthesized similarly to the method reported by Zhang, et al.^46^. For example, to prepare a composite sample with the 20 wt% of GO and 20 wt% of ZnO nanoparticles, the chitosan powder (0.75 g) was dissolved in 30 ml acetic acid solution (1.5% w/v) and the GO nanoparticles (0.25 g) was added to the solution. The solution was stirred at room temperature for 3 h followed by adding the ZnO nanoparticle (0.25 g), and the flask contents were mixed for 30 min. Afterward, the flask contents were poured into a Teflon-lined autoclave and the autoclave was placed inside an oven at the temperature of 120 °C for 12 h. Finally, the autoclave’s contents were cooled down to room temperature and the resulting composite was purified using deionized water and methanol followed by drying in an oven at 95 °C for 10 h. The general synthesis procedure of the CTS/GO/ZnO composite is illustrated in Fig. 2. Similar to the mentioned procedure, the CTS/GO/ZnO-10 wt% sample was prepared by using the chitosan powder (0.80 g), GO nanoparticles (0.10 g), and ZnO nanoparticle (0.10 g), also for the case of CTS/GO/ZnO-30 wt%, the chitosan powder (0.60 g), GO nanoparticles (0.45 g), and ZnO nanoparticle (0.45 g) were used.

### Adsorbent characterization

X-ray diffraction analysis (XRD, STOE STADIMP, Germany) was used to determine the crystalline structure, and phases of the samples under Cu Ka radiation, 40 kV voltage. Also, X'Pert HighScore Plus software was utilized for phase detection. To characterize the samples' textural properties and pore characteristics, nitrogen adsorption/desorption experiment was performed by utilizing an ASAP 2020 M analyzer. Fourier Transfer Infrared (FTIR) analysis was conducted using a PerkinElmer FTIR spectrometer to detect the samples' functional groups.

### Adsorption setup

**Figure 3:**
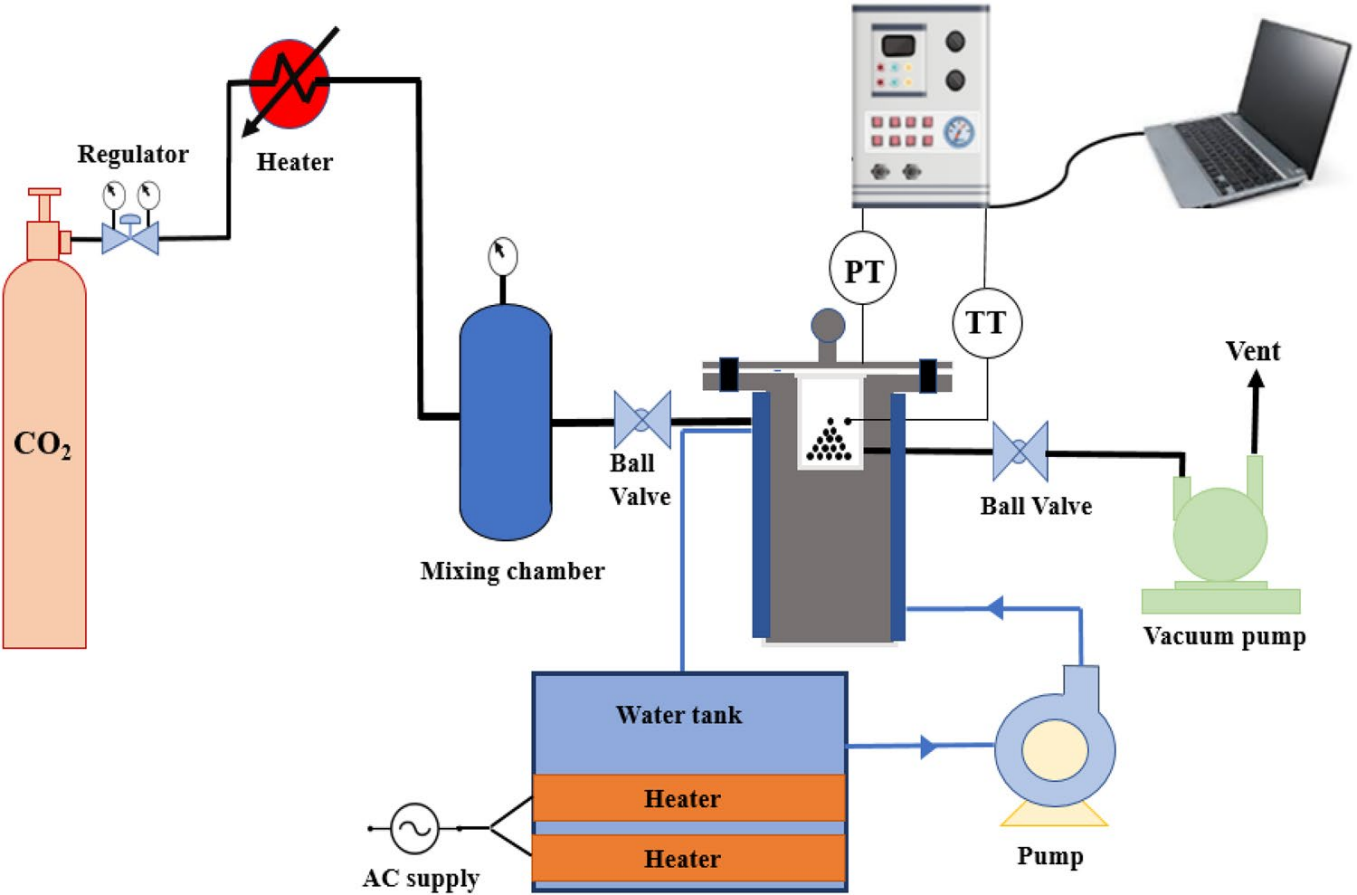
Experimental CO_2_ adsorption setup.

The CO_2_ adsorption capacity of the obtained solid adsorbents was measured by utilizing a volumetric setup which was illustrated in Fig. 3. As can be seen in Fig. 3, the pure CO_2_ gas is warmed up by utilizing an electrical heater followed by entering into a mixing chamber, and inside the chamber, both the pressure and the temperature of the CO_2_ gas equalize. Next, the gas is entered into the adsorption vessel and the gas will be contacted to the bulk powder of the adsorbent. The adsorption vessel consists of a stainless steel chamber with a total volume of 254 cm^3^, inner diameter of 6 cm, and height of 9 cm, which is sealed by an appropriate cap to eliminate the leak of the gas to the environment. The adsorption vessel’s temperature is adjusted at the set point temperature using a water jacket placed around the adsorption vessel. As soon as the CO_2_ gas is entered into the adsorption vessel, the CO_2_ uptake process will be started and the vessel’s features including the pressure and temperature and their corresponding adsorption time are collected. The CO_2_ adsorption capacity can be calculated by considering the CO_2_ gas’s mass differences between the initial and final adsorption time using Eqs. (1) and (2)^47^.

1 $$q=\frac{{m}_{i}-{m}_{f}}{W}=\left(\frac{V\times {\text{Mw}}}{R\times W}\right)\times \left({\left[\frac{P}{Z\times {\text{T}}}\right]}_{i}-{\left[\frac{P}{Z\times {\text{T}}}\right]}_{f}\right),$$

2 $$Z=1+\frac{B\times P}{R\times T},$$

Which m_i_ and m_f_ refer to the mass of the CO_2_ gas at the initial and final adsorption time. The subscripts i and f are related to the initial and final condition. Also, the terms including P is pressure, V is adsorption vessel volume, R is the global gas constant, T is temperature, M_w_ is CO_2_ molecular weight, W is adsorbent’s weight, and Z is gas compressibility factor. The term B is the second virial coefficient which can be calculated by using an empirical correlation for both polar and non polar gaseous system introduced by Tsonopoulos at 1974^48,49^. The mentioned correlation and its parameters are presented in Eqs. (3), (4) and (5)

3 $$B=\frac{R\times {T}_{c}}{{P}_{c}} ({F}^{\left(0\right)}{(T}_{r})+\upomega {F}^{\left(1\right)}{(T}_{r})),$$

4 $${F}^{\left(0\right)}{(T}_{r})=0.1445-\frac{0.330}{{T}_{r}}-\frac{0.1385}{{T}_{r}^{2}}-\frac{0.0121}{{T}_{r}^{3}}-\frac{0.000607}{{T}_{r}^{8}},$$

5 $${F}^{\left(1\right)}{(T}_{r})=0.0637+\frac{0.331}{{T}_{r}^{2}}-\frac{0.423}{{T}_{r}^{3}}-\frac{0.008}{{T}_{r}^{8}}.$$

### Response surface methodology

Recently, in the various fields of engineering, the design of experiment (DOE) has been used as an applicable tool that can correlate independent variables to the response regarding considering the variables' interactions with each other. DOE by utilizing response surface methodology (RSM) can be applicable to investigate and model the situations where a modeling’s response is influenced by some independent variables within a defined design space. Investigation of the variables' interaction concerning decreasing the number of experiments is considered a main advantage of the RSM technique. In this method, coefficients of a quadratic model represented in Eq. (6), are determined by fitting the experimental values on the mentioned equation, also the obtained model’s significance is evaluated through analysis of the variance (ANOVA) using some statistical criteria such as model’s P-value, F-value, and correlation coefficient (R^2^)^3,15^. The R^2^ value and absolute average relative error (AARE%) can be calculated using Eqs. (7) and (8), respectively^50^.

6 $$Y={\upbeta }_{0}+{\sum }_{i=1}^{n}{\upbeta }_{i }{x}_{i}+ {\sum }_{i=1}^{n}{\upbeta }_{ii }{x}_{ii}^{2}+ {\sum }_{\begin{array}{c}i=1\\ j>i\end{array}}^{n}{\upbeta }_{ij }{x}_{i}{x}_{j},$$

7 $$R^{2} = \frac{{(q^{\exp } - \overline{q}^{cal} )^{2} }}{{\sum\limits_{i = 1}^{N} {\left( {(q^{\exp } - \overline{q}^{cal} )^{2} + (q^{\exp } - q^{cal} )^{2} } \right)} }},$$

8 $$\% AARE = \left( {\sum\limits_{i = 1}^{N} {\left| {\frac{{q^{\exp } - q^{cal} }}{{q^{\exp } }}} \right|/N} } \right) \times 100,$$

where the term ${\upbeta }_{0}$ refer to the model’s constant, ${\upbeta }_{i}$ indicates linear effect, ${\upbeta }_{ii}$ refers to the quadratic effect, ${\upbeta }_{ij}$ is the interaction term, and n is equal to the variable’s count.

The RSM technique’ Box Behnken Design (BBD) is regarded as a successful strategy for correlating independent factors to response. In this method, the range of variables is separated into three levels: − 1, 0, and + 1, which are related to the interval’s lower bound, middle point, and upper bound, respectively^51^. In this study, the DOE was applied in the Design Expert software (version 11) by utilizing RSM-BBD approach to correlate the effect of independent variables including graphene oxide (GO) loading (wt.%), Zinc Oxide (ZnO) loading (wt.%), adsorption process’s temperature (T), and pressure (P) on the CO_2_ adsorption capacity of the GO-ZnO- chitosan composite adsorbent. The mentioned variables’ intervals are reported in Table 1.

**Table 1:** Independent variables levels in RSM-BBD modeling.

Independent variables				Levels		
Full name	Abbreviation	Unit	Type	− 1	0	1
GO loading	A	(wt%)	Numeric	10	20	30
ZnO loading	B	(wt%)	Numeric	10	20	30
Temperature	C	^°^C	Numeric	25	45	65
Pressure	D	bar	Numeric	1	5	9

## Results and discussion

### RSM results

#### Analysis of variance (ANOVA)

The ANOVA results of the CO_2_ uptake process using CTS/GO/ZnO composite and the impact of effective factors such as GO loading weight percent, ZnO loading weight percent, adsorption process temperature, and pressure are presented in Table 2. To determine the accuracy of the obtained RSM-based model within the design space, two statistical criteria including P-value and F-value are presented which refer to the significance of the obtained model or the model’s terms when the P-value is less than 0.05 and F-value is more than 1. Therefore, by considering the P-value of the model (less than 0.0001) in Table 2, it can be concluded the obtained model has enough precision within design space, also the P-values of the model's terms show that the terms including GO loading (wt%), temperature, and pressure are particularly significant and more effective than the term ZnO loading (wt%), in the obtained model. The model's F-value (132.65) is significantly higher than 1, which highlights the model's importance and the low likelihood that it is the result of noise (probability is less than 0.01%)^52^. The generated model is trustworthy because the R^2^ = 0.992 is higher than 0.8 and the differences between the predicted R^2^ = 0.954 and the adjusted R^2^ = 0.985 is less than 0.2. So, the developed predictive model can be applicable for designing industrial processes because the adequate precision value for signal-to-noise evaluation is 43.21, which is more than 4.

**Table 2:** ANOVA results of the CO_2_ uptake modeling based on RSM-BBD.

Source	Sum of squares	df	Mean square	F-value	p-value
Model	4.012E + 05	14	28,654.20	132.65	< 0.0001
A-GO (wt%)	9290.77	1	9290.77	43.01	< 0.0001
B-ZnO (wt%)	353.24	1	353.24	1.64	0.2218
C-temperature (°C)	22,794.56	1	22,794.56	105.52	< 0.0001
D-pressure	3.516E + 05	1	3.516E + 05	1627.52	< 0.0001
AB	320.41	1	320.41	1.48	0.2434
AC	4590.06	1	4590.06	21.25	0.0004
AD	21.16	1	21.16	0.0980	0.7589
BC	99.16	1	99.16	0.4591	0.5091
BD	248.06	1	248.06	1.15	0.3020
CD	848.93	1	848.93	3.93	0.0674
A^2^	14,323.70	1	14,323.70	66.31	< 0.0001
B^2^	1949.31	1	1949.31	9.02	0.0095
C^2^	1107.98	1	1107.98	5.13	0.0399
D^2^	3267.94	1	3267.94	15.13	0.0016
Residual	3024.24	14	216.02		
Lack of fit	3024.24	9	336.03		
Pure error	0.0000	5	0.0000		
Cor total	4.042E + 05	28			

R^2^ = 0.992, R^2^_pred_ = 0.954, R^2^_adj_ = 0.985, Adequate precision = 43.21.

#### CO_2_ uptake model based on the RSM-BBD approach

**Figure 4:**
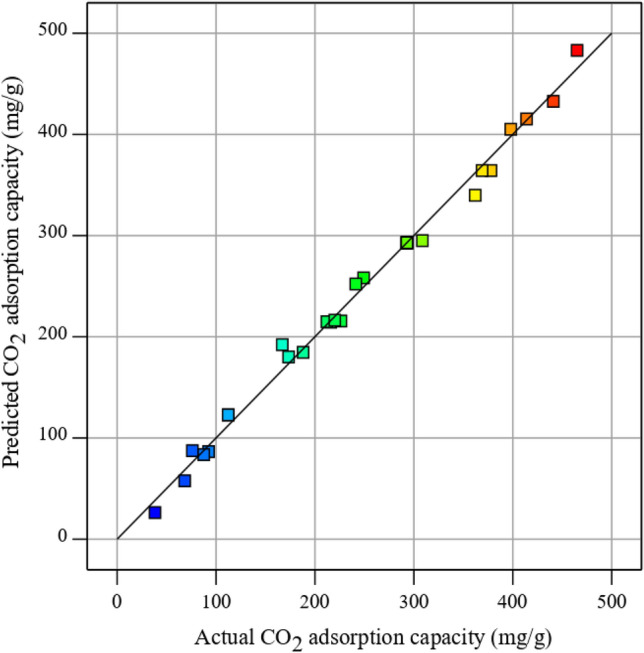
Predicted vs. experimental quantity of CO_2_ uptake capacity.

To correlate the effect of the mentioned parameters including GO loading weight percent, ZnO loading weight percent, adsorption process temperature, and pressure on the CO_2_ uptake capability of the CTS/GO/ZnO composite, A quadratic model, obtained from the RSM-BBD method, is presented in Eq. (9). Also, comparison between the CO_2_ adsorption quantities, predicted using RSM-based model and the experimental values is illustrated in Fig. 4. According to this figure, the provided semi-empirical model's superior precision can be proved by considering the sufficient centralization of the predicted and the experimental values of the CO_2_ adsorption capacity over the diagonal line.

9 $${{\text{q}}}_{{{\text{CO}}}_{2}}= -485.343+31.4217\times {\text{A}}+10.1054\times {\text{B}}+5.582\times {\text{C}}+60.5028\times {\text{D}}-0.0895\times {\text{AB}}-0.169375\times {\text{AC}}+0.0575\times {\text{AD}}-0.0313535\times {\text{BC}}+0.196875\times {\text{BC}}-0.167471\times {\text{CD}}-0.473497\times {{\text{A}}}^{2}-0.182393\times {{\text{B}}}^{2}-0.0336579\times {{\text{C}}}^{2}-1.40772\times {{\text{D}}}^{2}.$$

#### Effect of input parameters on the CO_2_ uptake capability of CTS/GO/ZnO composite

**Figure 5:**
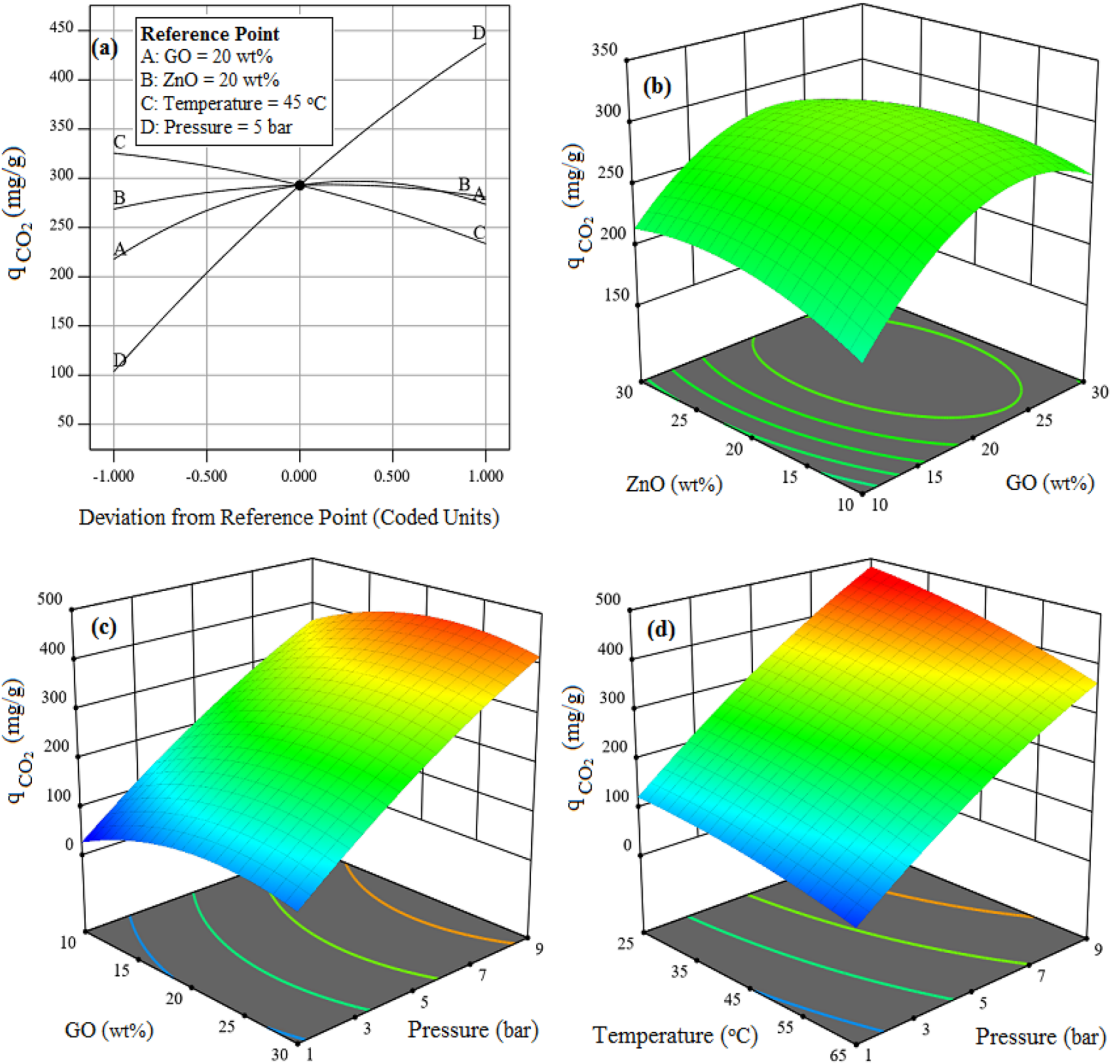
CO_2_ adsorption capacity dependency to the (**a**) all factors, (b) GO and ZnO loading weight percent at T = 45 °C and P = 5 bar, (**c**) GO loading weight percent and pressure at = 45 °C and ZnO = 20 wt%, and (**d**) temperature and pressure when GO = 20 wt% and ZnO = 20 wt%.

To investigate the effect of input factors on the CTS/GO/ZnO composite’s CO_2_ adsorption capability, perturbation plot is prepared and shown in Fig. 5. In this figure, the A, B, C, and D curves correspond to the factors namely GO loading weight percent, ZnO loading weight percent, temperature, and pressure, respectively. According to the Fig. 5a, an increase in CO_2_ uptake capability of the CTS/GO/ZnO composite can be observed by gaining the GO and ZnO nanoparticles loading on the chitosan bio adsorbent up to 22.5 wt% and 18.3 wt%, respectively, while increasing the loading more than mentioned values causes a gradual reduction in CO_2_ adsorption capacity of the solid sorbent. The CTS/GO/ZnO composite’s CO_2_ adsorption improvement can be attributed to the increasing surface area of the composite sample in comparison with pristine samples such as chitosan bio adsorbent, GO, and ZnO nanoparticles. Also, the oxygen-rich nature of the GO and ZnO samples and also the presence of various electrons withdrawing functional groups such as –NH_2_, –COOH, –NO_2_, and –OH in the GO sample’s structure, make these nanoparticles a promising candidate for CO_2_ uptake applications^53^. The mentioned functional group’s dispersion on the surface of the chitosan sample causes increasing heterogeneity of the composite sample, resulting in gaining the CO_2_ uptake capability of the CTS/GO/ZnO composite through improving dipole-quadropole interaction between the adsorbent’s surface and CO_2_ molecules^24^. However, increasing the GO and ZnO nanoparticles loading value more than the critical quantity (GO = 22.5 wt% and ZnO = 18.3 wt%), causes a decrease in the CO_2_ capture capability of the composite sample. It can be related to the pore filling and decreasing adsorbent’s pore width and pore blocking effect in the excess loading values of the GO and ZnO nanoparticles^54^. The CO_2_ adsorption capacity in CTS/GO/ZnO is influenced by surface area and pore volume. A larger surface area allows more CO_2_ interaction and adsorption, with materials like GO and ZnO having high surface areas. Pore volume provides space for CO_2_ trapping, increasing the adsorption capacity. The composite nature of CTS/GO/ZnO leads to synergistic effects, where their properties complement each other for higher CO_2_ adsorption. As shown in the Fig. 5a, increasing the adsorption process’s pressure causes a sharp enhancement in the CO_2_ uptake capability of the CTS/GO/ZnO composite. It can be attributed to the improved mass transfer of CO_2_ molecules into the previously inaccessible cavities as well as the decrease in the desorption of the captured CO_2_ molecules^55^. Considering a decreasing behavior of the CO_2_ adsorption by increasing the process’s temperature, it can be concluded the CO_2_ uptake reduction may be related to the physical adsorption of the CO_2_ molecules on the surface of the composite sample, also an increase in temperature increases the movement of absorbed CO_2_ molecules, which leads to improved desorption of CO_2_ molecules at higher temperatures^24^.

To better show the effect of the mentioned factors and their interactions on the CO_2_ adsorption capacity of the CTS/GO/ZnO sample, three-dimensional plots are provided and shown in Fig. 5c to Fig. 5d. Increasing the presence of GO and ZnO in Fig. 5b is shown to boost CO_2_ adsorption. However, it's worth noting that an excessive amount of these materials can diminish the overall CO_2_ adsorption capacity. This happens because, past a certain point, the surface sites on these materials get filled up or blocked. When there's an excess of GO and ZnO, it can impede these materials' ability to efficiently capture and retain CO_2_ molecules. Instead of enhancing adsorption, an overabundance of material can create obstacles that restrict the interaction between CO_2_ and the available adsorption sites, resulting in a decrease in the overall adsorption capacity. Therefore, maintaining an optimal balance in the loading levels of these materials is crucial for achieving the best CO_2_ adsorption performance. As shown in Fig. 5b, an increase in pressure at the optimum loading of GO in composite can increase CO_2_ adsorption. This occurs because higher pressure provides a driving force for CO_2_ molecules to be more readily attracted to the adsorption sites on the GO surface. With increased pressure, there are more CO_2_ molecules in the gas phase, and they are more likely to interact with and be adsorbed by the available sites on the GO material. As a result, the overall CO_2_ adsorption capacity is enhanced under these conditions, making it an effective strategy for improving the adsorption process.

Increasing in pressure and decreasing in temperature can increase CO_2_ adsorption capacity. This phenomenon occurs because higher pressure and lower temperature conditions favor the formation of stronger interactions between CO_2_ molecules and the adsorption sites on the material's surface. At higher pressure, there are more CO_2_ molecules available in the gas phase, increasing the chances of these molecules coming into contact with and being adsorbed by the adsorption sites. Additionally, lower temperatures reduce the kinetic energy of CO_2_ molecules, making them more likely to be captured and held by the adsorption sites, as their movement is slowed down. These combined effects result in a higher adsorption capacity for CO_2_, making it a more efficient process as shown in Fig. 5d. According to these figures, the highest CO_2_ adsorption capacity of the CTS/GO/ZnO composite can be obtained at the maximum pressure, minimum process’s temperature, and the middle ranges of GO and ZnO nanoparticles loading weight percent.

#### CO_2_ capture process optimization

Industrial use of the CTS/GO/ZnO composite requires fine-tuning the operational parameters to achieve the highest CO_2_ uptake capacity. The CO_2_ adsorption optimization performed here was accomplished with the help of the optimization module of the Design Expert software. To determine the maximum uptake capacity of the composite, the effective parameters including GO loading, ZnO loading, temperature, and pressure were set as 'in range' and the adsorption capacity was set to 'maximize' followed by running optimization. Table 3 reports the optimum condition with the highest desirability. By conducting the CO_2_ capture experiment under the obtained conditions, the RSM-BBD approach's optimized CO_2_ adsorption capability was experimentally verified. As a result, the experimental CO_2_ uptake capacities of 479.08, 476.33, 475.08, and 479.19 mg/g and an insignificant AARE of about 1.42% was obtained after four replications of the adsorption test under optimum conditions.

**Table 3:** The result of CO_2_ uptake process optimization using RSM-BBD.

Factors	Target	Lower limit	Upper limit	Optimum value
GO (wt%)	In range	10	30	23.8
ZnO (wt%)	In range	10	30	18.2
Temperature (°C)	In range	25	65	30.1
Pressure (bar)	In range	1	9	8.6
CO_2_ adsorption capacity (mg/g)	Maximize			470.43

### Adsorbents characterization

**Figure 6:**
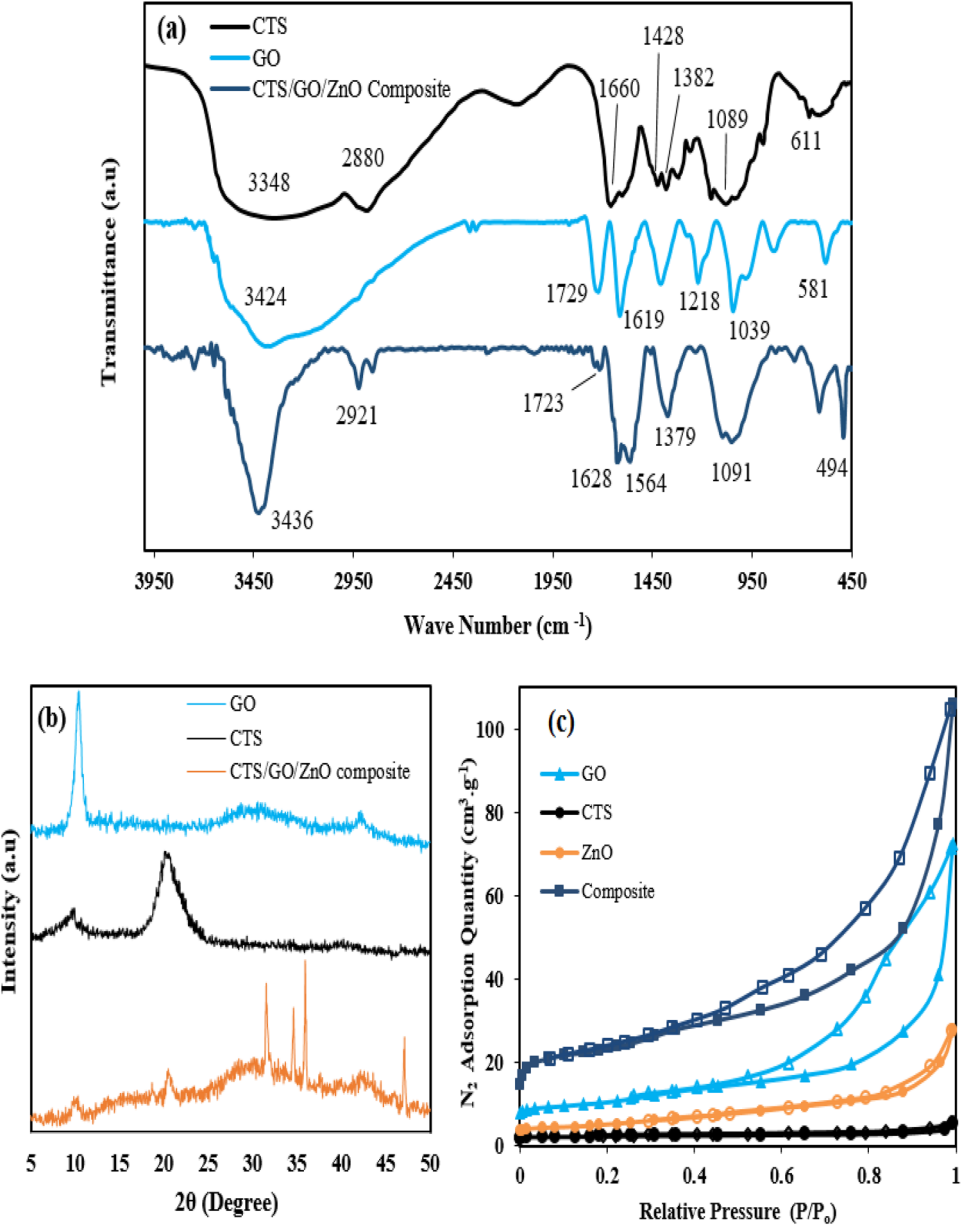
Chitosan, GO, and CTS/GO/ZnO Samples characterization based on (**a**) FTIR spectra between 450 and 4000 cm^–1^, (**b**) XRD pattern at 5 < 2θ < 50, and (**c**) N_2_ adsorption–desorption isotherm at 77.3 K.

The FTIR spectra of the samples namely chitosan, GO, and CTS/GO/ZnO (GO = 23.8 wt%, ZnO = 18.2 wt%) are displayed in Fig. 6a. In the chitosan FTIR spectra, a band allocated at 3348 cm^-1^ is correspond to the O–H and N–H stretching vibration of hydroxyl group and primary amine group (NH_2_), also the characteristic peaks allocated at 2880, 1660, and 1089 cm^–1^ are attributed to the C–H stretching vibration of CH and CH_2_, N–H bending vibration of the NH_2_, and C–O stretching vibration of the hydroxyl moiety, respectively. Moreover, two peaks at 1428 1382 cm^–1^ refer to the deformation vibration of the N–H bond in the NH_2_^44,56^. The FTIR spectrum of the GO sample reveals peaks at 3424, 1729, 1619, 1218, and 1039 cm^–1^ which corresponds to the O–H stretching vibration of carboxylic acid or hydroxyl functionalities, C=O stretching vibration of carboxylic acid, C=C bond’s vibration of unoxidized graphite moiety, C–O stretching vibration, and C–O–C stretching vibration of the epoxy group^57^. Considering the FTIR spectra of the CTS/GO/ZnO sample and GO sample, it can be observed the intensity of the C=O bond (1723 cm^–1^) is reduced, which refers to the chemical bonding between carboxylic acid and amine functionalities and formation of the amide group. A strong peak at 3436 cm^–1^ is attributed to the stretching vibration of O–H, N–H, and hydrogen bonding of polysaccharide molecules, also the peak at 1564 cm^–1^ is ascribed to the N–H vibration of the amide group. Moreover, a band at 494 cm^–1^ is related to the Zn–O bond of ZnO nanoparticles of the CTS/GO/ZnO composite^46,57^. The results of XRD analysis of the GO, chitosan, and CTS/GO/ZnO composite (GO = 23.8 wt% and ZnO = 18.2 wt%) are illustrated in Fig. 6b. According to the GO’s XRD pattern a sharp diffraction peak at 2θ = 10.9^◦^ is related to the crystalline nature of the GO^35^. The XRD pattern of the chitosan sample exhibited two diffracted peaks near the 2θ = 9.9^◦^ and 2θ = 20.8°, which are attributed to the crystalline structure of commercial chitosan sample (JCDPS no.39-1894)^58^. Considering the XRD pattern of the CTS/GO/ZnO composite, it can be observed that some characteristic peaks have appeared at the 2θ quantities equal to 31.6°_,_ 34.7°, 36.1° and 47.5°. These peaks are attributed to the ZnO nanoparticle’s crystal plains of 100, 001, 101, and 102, respectively (JCDPS no.36-1451)^46^. Moreover, the intensity of the peaks located at 2θ = 10.4° and 2θ = 20.5^◦^ in the composite sample’s XRD pattern are weaker than the peaks of the GO and chitosan pristine samples, therefore it can be concluded the crystallinity of the composite sample is reduced and the final structure turned into amorphous structure^44^.

**Figure 7:**
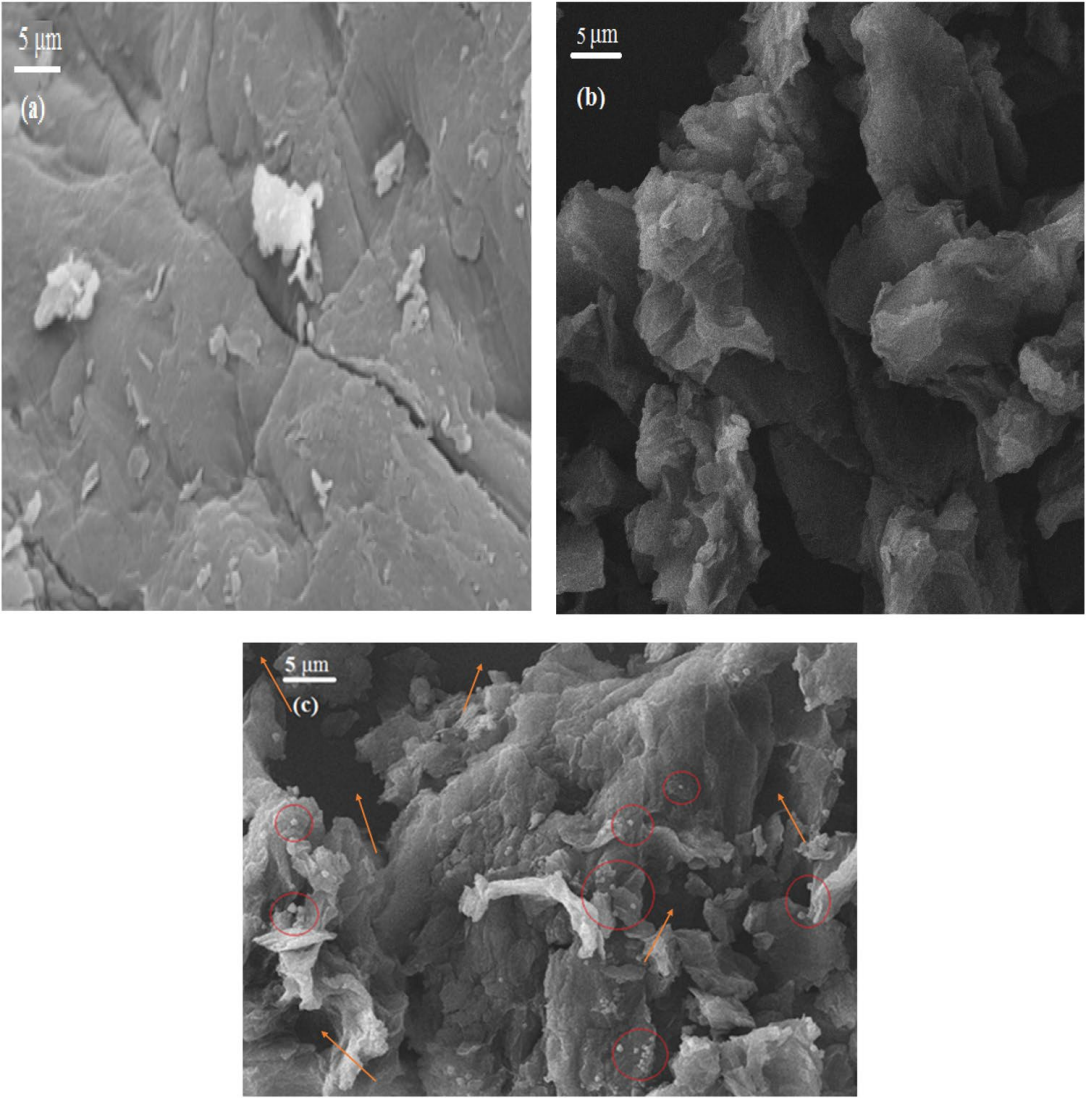
SEM image of (**a**) Chitosan, (**b**) GO, and (**c**) CTS/GO/ZnO composite.

The charactrization properties of the mentioned samples were investigated by conducting a Nitrogen adsorption–desorption experiment at 77.3 K. The isotherm plots of the N_2_ adsorption–desorption are illustrated in the Fig. 6c and the morphological characteristics of the samples are presented in Table 4. Based on the findings of the Fig. 6c, all samples exhibit IV type isotherm (based on IUPAC isotherm classification) and H2 hysteresis loop. Considering isotherm plots, a minor quantity of the N_2_ was adsorbed by the GO, ZnO, and chitosan sample at a relative pressure of less than 0.2, therefore these samples are rarely constructed by microspores. Meanwhile, the composite sample indicated more quantity of N_2_ adsorption at the mentioned relative pressure range, so it can be concluded the micropore volume of the composite sample is much more than other samples^35,59^. Considering the hysteresis loop presence in the samples isotherm plots at the relative pressure between 0.2 and 0.8, it can be noticed that all samples especially GO, and the CTS/GO/ZnO samples formed by meso-pores, also the hysteresis loop at the relative pressure higher than 0.8 indicate the existence of macro-pores and intraparticle cavities^49^. As a result, the surface area of a composite of GO, ZnO, and CTS is enhanced due to their synergistic effects. GO, being a two-dimensional carbon material, provides a large surface area for adsorption and increased porosity, while ZnO contributes to surface roughness and provides active sites for chemical interactions. Chitosan acts as a binder, promoting the dispersion of GO and ZnO, further increasing the available surface area. This unique combination maximizes the accessible surface for CO_2_ adsorption. SEM images of the chitosan, GO, and CTS/GO/ZnO composite are shown in Fig. 7. Based on the SEM images, the surface porosity of the composite sample is more than the GO and chitosan samples, also the composite sample is formed from cavities with a narrower pore size compared with GO sample. The area circled as red color in the Fig. 7c indicate ZnO nanoparticles were successfully dispersed and loaded on the surface of the composite sample. In addition, sections marked in the Fig. 7c with arrows indicate the porosity for CO_2_ adsorption.

**Table 4:** Characteristics of all samples based on N_2_ adsorption–desorption analysis.

Sample	S_BET_^a^ (m^2^/g)	MPV^b^ (cm^3^/g)	PV^c^ (cm^3^/g)	APD^d^ (nm)
GO	36.53	< 0.0001	0.043	4.73
Chitosan	7.76	< 0.0001	0.010	5.15
ZnO	17.91	< 0.0001	0.021	4.69
CTS/GO/ZnO composite (GO = 23.8 wt%, ZnO = 18.2 wt%)	78.75	0.009	0.056	2.84

^a^BET Surface area, ^b^Micropore volume, ^c^Total pore volume, ^d^Average pore diameter (measured using BET surface area and 4V/A equation).

### Adsorption isotherms

To effectively design adsorption systems, isotherm modeling may provide relevant data on the adsorption process mechanism. Such models explain the nature of the interactions and reactions that occur between the solid sorbent’s surface and adsorbate's molecules during gas capture processes. The current study used isotherm models such as Langmuir, Freundlich, Dubbin-Radushkevich (D-R), and Temkin as two-parameter models to explain the adsorption process. The aforementioned models are highlighted in Eqs. (10), (11), (12), (13), respectively^60^.

10 $${\text{Langmuir:}}\,\, {q}_{e}=\frac{{q}_{l} {K}_{l}{ P}_{e}}{1+{K}_{l}{ P}_{e}},$$

11 $${\text{Freundlich:}}\,\, {q}_{e}={k}_{F}{P}_{e}^{1/{n}_{F}},$$

12 $${\text{Temkin:}}\,\, {q}_{e}=B Ln {A}_{T}+B Ln {P}_{e}, B=\left(\frac{RT}{{b}_{T}}\right),$$

13 $${\text{Dubinin}}-{\text{Radushkevich:}}\,\, {q}_{e}={q}_{D}{\text{exp}}\left(-{\lambda \omega }^{2}\right),$$

where q_e_ and P_e_ refer to equilibrium CO_2_ uptake capacity (mg.g^–1^) and equilibrium pressure (bar). Other terms such as ${q}_{l}$ is maximum uptake capacity (mg.g^–1^) based on the Langmuir model, K_l_ (bar^–1^) is Langmuir model’s constant, K_F_ (mg.g^–1^.bar ^1/n^) and n_F_ are Freundlich model’s constants. In the Temkin model, the term A_T_ (L.mol^–1^) is the model’s constant and B is the virial equation of the state’s first coefficient ($B=\left(\frac{RT}{{b}_{T}}\right), {b}_{T}=(J.{mol}^{-1})$), the terms $\lambda$ (mol^2^.J^–1^) and $\omega$ (J.mol^–1^) in the D-R model refer to the model’s constant and Polanyi potential, respectively^60^.

To perform isotherm modeling, a new composite sample was prepared according to the optimum GO and ZnO nanoparticles loading weight percent (GO = 23.8 wt% and ZnO = 18.2 wt%), followed by conducting CO_2_ adsorption tests at the different pressures between 1 to 9 bar and the temperature of 298 K, 308 K, 318 K, and 328 K.

**Figure 8:**
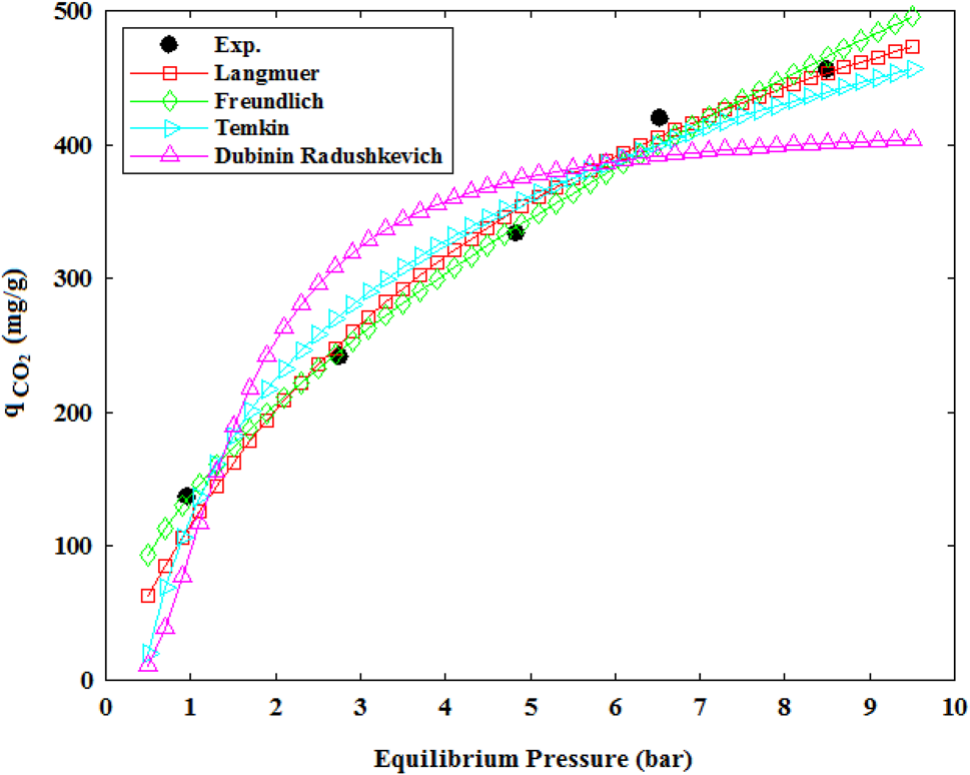
The fitted plots of CO_2_ adsorption isotherm at the temperature of 25 °C.

Next, the mentioned models were fitted on the equilibrium adsorption data and the model’s terms were obtained which are reported in Table 5, also the fitted curves of the isotherm models at 298 K are displayed in Fig. 8. Based on the Table 5 findings, a descending order of the Langmuir model’s constant (${q}_{l}$) by increasing temperature, proves the exothermic nature of CO_2_ uptake process. In addition, by considering the K_F_ value of the Freundlich model that refers to the tendency of the adsorbate molecule to be adsorbed by solid sorbent, it can be observed increasing temperature caused a gradual decrease in the K_F_ value, so the CO_2_ adsorption by CTS/GO/ZnO composite occurred via physisorption mechanism dominantly^61^. Also, a favorable CO_2_ capture process is reflected by the Freundlich model’s parameter (n_F_), which lies between 1 and 2. Furthermore, the D-R model’s constant ($\omega$) stands for the free energy of adsorption, a physisorption mechanism is indicated by the $\omega$ values below 8 kj/mol, while chemisorption is indicated by the $\omega$ values between 8 and 16 kj/mol. Thus, the $\omega$ values below 8 kj/mol confirm the previously established physical adsorption of CO_2_ molecules^62^. As a result of comparing different isotherm models, the Freundlich model was chosen as the most appropriate model with the highest R^2^ value. Therefore, it can be concluded that the surface of CTS/GO/ZnO composite is heterogeneous, and multi-layer CO_2_ adsorption is performed on the adsorbent’s surface. A high Freundlich constant shows that the composite has a high adsorption capacity, while a low exponent means a more linear adsorption isotherm^49^.

**Table 5:** Parameters of the isotherm models at the temperature of 298 K, 308 K, 318 K and 328 K.

Model	Parameters	Temperature			
		298 K	308 K	318 K	328 K
Langmuir	${q}_{l}$	737.76	710.06	677.36	658.61
	${K}_{l}$	0.1878	0.1750	0.1596	0.1387
	R^2^	0.992	0.989	0.996	0.997
Freundlich	${K}_{F}$	138.63	126.95	110.15	94.77
	${n}_{F}$	1.76	1.736	1.661	1.588
	R^2^	0.996	0.995	0.996	0.997
Temkin	$\frac{RT}{{b}_{T}}$	148.32	132.55	132.65	121.33
	${A}_{T}$	2.283	2.522	2.008	1.948
	R^2^	0.981	0.972	0.985	0.983
Dubinin-Rudushkevich	${q}_{D}$	416.19	378.79	362.88	368.16
	$\lambda$	0.492	0.395	0.535	0.999
	$\omega$	1.008	1.124	0.966	0.708
	R^2^	0.908	0.886	0.926	0.970

### Kinetic modeling

**Figure 9:**
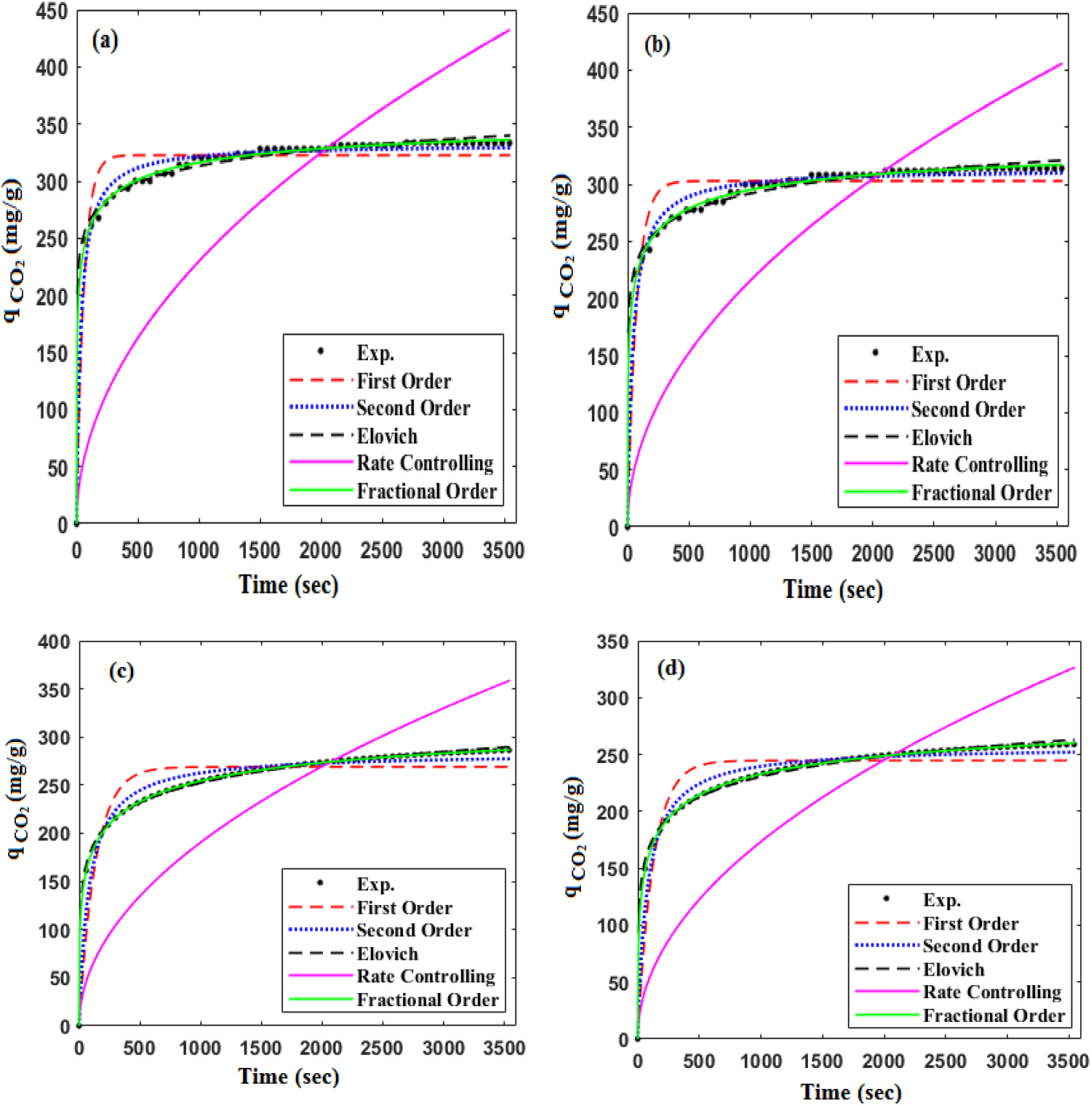
Kinetic models fitting on the experimental data of the CO_2_ adsorption at the pressure of 5 bar and temperature of (**a**) 298 K, (**b**) 308 K, (**c**) 318 K, and (**d**) 328 K.

Generally, investigation of an adsorption process kinetic can provide some useful insights about the rate of adsorption which significantly affects adsorption efficiency. Typically, kinetic models are employed to determine the gas phase’s retention time and adsorption rate, which are practical for industrial process design purposes such as fixed bed columns. Therefore, to investigate the CO_2_ adsorption kinetic, common kinetic models such as first order, second order, Elovich, Rate Controlling, and Fractional order models were applied. The aforementioned kinetic models are introduced in Eqs. (14), (15), (16), (17) and (18), respectively. The experimental CO_2_ adsorption data were collected by utilizing the newly synthesized CTS/GO/ZnO composite (GO = 23.8 wt% and ZnO = 18.2 wt%) at the pressure of 5 bar and the temperature 298 K, 308 K, 318 K, and 328 K. The results of kinetic modeling and the model’s parameters are summarized in Table 6, also the curves of the kinetic models were plotted and illustrated in Fig. 9.

14 $$\mathrm{First \,\,order:}\,\, {q}_{t}={q}_{e}\left(1-{e}^{{k}_{1}t}\right),$$

15 $$\mathrm{Second \,\,order:}\,\, {q}_{t}=({q}_{e}^{2} {k}_{2}t)/( 1+{q}_{e}{k}_{2}t),$$

16 $${\text{Elovich:}}\,\, {q}_{t}=\beta {\text{ln}}\left(\alpha \beta \right)+ \beta {\text{ln}}\left(t\right),$$

17 $$\mathrm{Rate \,\,controlling:}\,\, {q}_{t}= {k}_{c}{t}^{0.5},$$

18 $$\mathrm{Fractional \,\,order:}\,\, {q}_{t}={q}_{e}-{\left[\frac{\left(n-1\right)}{m} {k}_{n} {t}^{m}+{q}_{e}^{\left(1-n\right)}\right]}^{\left(1/\left(1-n\right)\right)}.$$

where the terms including k_1_, k_2_, k_c_, and k_n_ are the constant of their corresponding kinetic models^63^. The first-order model assumes that the rate of adsorption is proportional to the difference between the saturation concentration and the quantity of the adsorbed constituent. The growing influence of chemical adsorption on the adsorption process is reflected in a decline in the R^2^ quantity of the first-order model, as reported in Table 6^64^. The rate controlling model suggests that the rate of the adsorption is affected by intraparticle diffusion. Increasing the R^2^ value of this model at higher temperatures suggests that diffusion is the rate-controlling mechanism^65^. Table 6 shows that the fractional order kinetic model successfully correlates the CO_2_ adsorption capacity to the adsorption time, as measured by the correlation coefficient (R^2^). A more accurate representation of the adsorption process that departs from integer order kinetics is provided by the latter model. It takes into account factors like surface heterogeneity, multilayer adsorption, and interactions between adsorbate molecules and adsorbent surface, all of which add to the adsorption process’ complexity^66^.

**Table 6:** The parameters of the kinetic models at the pressure of 5 bar.

Model	Parameters	T = 298 K	T = 308 K	T = 318 K	T = 328 K
First order	${q}_{e}$	322.70	302.83	268.97	244.74
	${k}_{1}$	0.01714	0.01368	0.00738	0.00793
	R^2^	0.945	0.936	0.922	0.926
Second order	${q}_{e}$	332.39	313.56	283.65	257.34
	${k}_{2}$	0.00009	0.00008	0.00004	0.00005
	R^2^	0.986	0.984	0.980	0.982
Fractional order	${q}_{e}$	351.74	336.76	318.13	284.39
	${k}_{n}$	0.00005	0.0018	0.0165	0.0241
	m	0.7001	0.4510	0.344	0.333
	n	2.272	1.668	1.230	1.180
	R^2^	0.999	0.998	0.999	0.999
Rate controlling	${k}_{c}$	7.265	6.814	6.024	5.485
	R^2^	0.678	0.714	0.825	0.809
Elovich	$\alpha$	128.49	14.52	0.159	0.287
	$\beta$	21.139	22.950	29.79	25.85
	R^2^	0.980	0.980	0.997	0.994

### Adsorption thermodynamic

**Figure 10:**
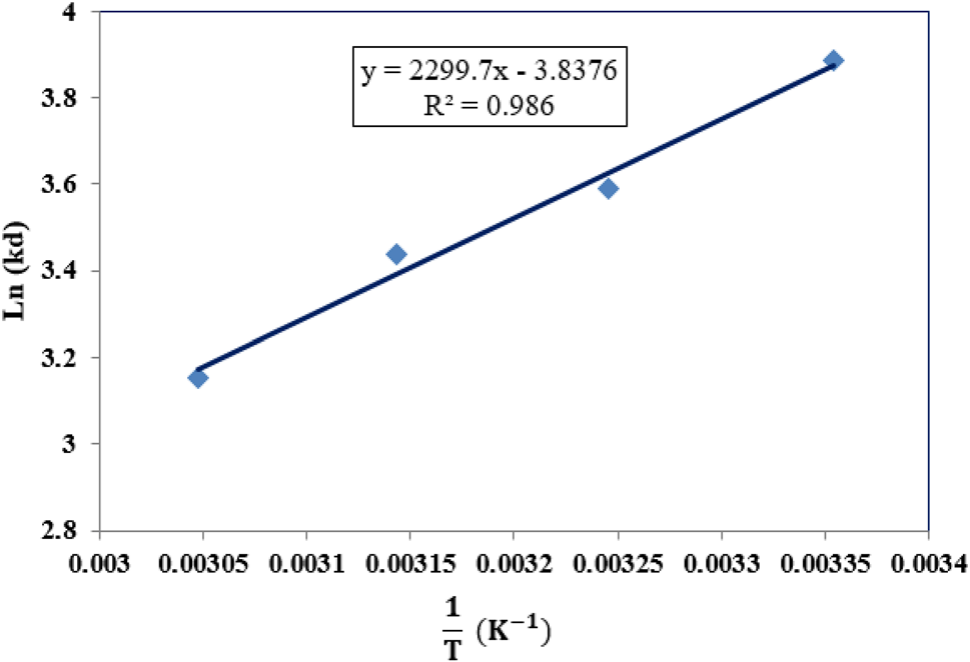
Van’s Hoff plots of the CO_2_ adsorption process at the pressure of 5 bar.

Thermodynamic analysis of the CO_2_ uptake process was applied to determine parameters such as enthalpy changes (ΔH), Gibbs free energy changes (ΔG), and entropy changes (ΔS). Using Eq. (19), the distribution factor (K_d_) was calculated at a constant pressure of 5 bar and the temperatures of 298 K, 308 K, 318 K, and 328 K. Van’t Hoff plot, shown in Fig. 10, was prepared through plotting the obtained K_d_ values versus their correspond reverse temperature ($\frac{1}{{\text{T}}})$. As presented in Eq. (20), the van’t Hoff plot’s slope and intercept refer to the enthalpy change (${\mathrm{\Delta H}}^{0}$) and entropy change (${\mathrm{\Delta S}}^{0}$), respectively. The ${\mathrm{\Delta G}}^{0}$ of the process can be measured using Eq. (21). The thermodynamic parameters of the CO_2_ adsorption process are reported in Table 7.

19 $${K}_{d}=\Delta P\times \frac{V}{w},$$

20 $${\text{ln}}\left({K}_{d}\right)=\frac{{\mathrm{\Delta S}}^{0}}{R}- \frac{{\mathrm{\Delta H}}^{0}}{RT},$$

21 $${\mathrm{\Delta G}}^{0}={\mathrm{\Delta H}}^{0}-T{\mathrm{\Delta S}}^{0},$$

where $\Delta P$ is the adsorption vessel’s pressure difference during the process, V is the vessel’s volume, W is defined as the adsorbent mass, and R refers to the gas constant (8.314 J.mol^–1^.K^–1^)^67^. According to the table, the ${\mathrm{\Delta H}}^{0}$ value (− 19.21 kJ/mol) reflects the physical adsorption of the CO_2_ molecules on the CTS/GO/ZnO composite regarding the heat releases quantity less than 20 kJ/mol for physisorption mechanism and heat releases more than 40 kJ/mol for chemisorption mechanism^68^. In addition, significant information about the randomized or organized interaction between the gas phase and the solid sorbent can be derived from the adsorption ${\mathrm{\Delta S}}^{0}$ value. A positive ${\mathrm{\Delta S}}^{0}$ value indicates that the adsorption is taken place more random, whereas negative ${\mathrm{\Delta S}}^{0}$ value proves less randomized adsorption. Considering the negative value of ${\mathrm{\Delta S}}^{0}$ (− 0.032 kJ/mol K), it can be inferred that the gas–solid interface is less random. The negative values of the ${\mathrm{\Delta G}}^{0}$ at different temperatures highlight the favorability of the CO_2_ adsorption process and also the processes occurred spontaneously^69^.

**Table 7:** Thermodynamic parameters of the CO_2_ adsorption process.

Sample	${\mathrm{\Delta H}}^{0}$(kJ/mol)	${\mathrm{\Delta S}}^{0}$(kJ/mol K)	${\mathrm{\Delta G}}^{0}$(kJ/mol)			
			298 K	308 K	318 K	328 K
CTS/GO/ZnO composite	− 19.121	− 0.032	− 9.608	− 9.288	−	− 8.650

### CTS/GO/ZnO composite renewability

**Figure 11:**
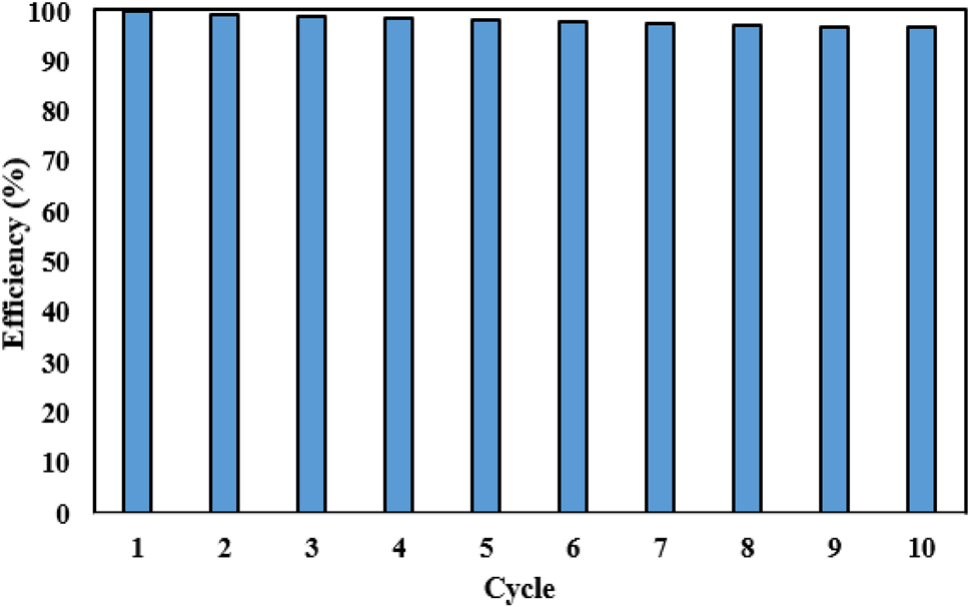
CTS/GO/ZnO composite recycling efficiency after ten cycle.

As the most important economic aspect for the development of new solid adsorbents, the recycling and reusability of the adsorbent should be investigated to further evaluate the feasibility of CO_2_ adsorption in an industrial scale. To investigate the stability and renewability of the resulting composite, ten cycles of the CO_2_ adsorption process were conducted at a pressure of 5 bar and temperature of 45 °C. In each cycle, the spent adsorbent was recycled at the temperature of 90 °C in a vacuum oven for 2 h. The composite’s efficiency in CO_2_ adsorption after ten cycles is plotted and illustrated in Fig. 11. According to this figure, minor losses of about 4.35% in the adsorbent efficiency for capturing CO_2_ can be observed after ten cycles. Therefore, the obtained composite can be used as a promising candidate for CO_2_ capture applications.

### Comparison between the current work and similar research

In this section, the CO_2_ uptake performance of the optimum CTS/GO/ZnO composite is compared with similar studies in this area. The results of the research on CO_2_ adsorption by utilizing GO, chitosan, and composite adsorbent and their corresponding operational conditions are reported in Table 8. According to this table, the synthesized CTS/GO/ZnO composite exhibits high CO_2_ adsorption capacity in comparison with other adsorbents, therefore it can be used as a promising adsorbent with the most efficiency for industrial scale CO_2_ capture applications.

**Table 8:** Comparison of the resulting CTS/GO/ZnO composite with similar adsorbents in capturing CO_2_.

Adsorbent	Surface area (m^2^/g)	Temperature (K)	Pressure (bar)	q (mg/g)	Refs
CTS	0.31	298	5	2.2	^70^
CTS/TPPS	26.75	298	5	37.41	^70^
CTS/GO film	6.67	298	4.6	44.67	^71^
CTS/GO aerogel	33.33	298	1	11.35	^35^
IFGO	190	273	3	94.16	^72^
CTS/4-formyltriphenylamine	52.78	298	5	59.37	^73^
ZY/CTS	22.53	298	4.6	51.48	^74^
CTS/ZIF-8	626.8	298	1	43.56	^75^
CTS/SiO_2_	621	298	1	193.16	^76^
CTS/PEI	69.13	313	0.15	101.2	^77^
CTS/PVA	–	298	–	7.92	^78^
Calcined CTS/GO-20%	412	298	1	182.6	^24^
CTS/GO/ZnO composite	78.75	298	1	141.32	This work
CTS/GO/ZnO composite	78.75	298	5	333.44	This work

## Conclusion

In this study, some types of the CTS/GO/ZnO composite with different loading weight percent of the GO and ZnO nanoparticles were prepared, and the CO_2_ adsorption experiments were conducted using resulting solid adsorbents. RSM-BBD technique was applied to investigate the effect of some parameters including GO loading, ZnO loading, temperature, and pressure on the CO_2_ adsorption capability of the adsorbents. Perturbation plots and three-dimensional response surfaces, prepared based on the RSM method, indicate that increasing the GO and ZnO loading weight percent up to 22.5 wt% and 18.3 wt% causes an improvement in the CO_2_ adsorption capacity of the composite adsorbent, meanwhile increasing the GO and ZnO loading more than mentioned quantity caused decreasing in the CO_2_ adsorption capacity due to pore blocking effect of the excess GO and ZnO nanoparticles. Optimization based on the RSM-BBD approach was conducted and the optimum structure of the CTS/GO/ZnO composite was obtained with the GO loading equal to 23.8 wt%, and ZnO loading equal to 18.2 wt%. The optimum composite characterization was performed using BET, XRD, FTIR, and SEM analysis. The BET analysis of the optimum CTS/GO/ZnO composite indicated that the surface area of the composite sample (SA_BET_ = 78.75 m^2^/g) is much more than pristine samples such as GO (SA_BET_ = 36.53 m^2^/g), chitosan (SA_BET_ = 7.76 m^2^/g), and ZnO (SA_BET_ = 17.91 m^2^/g). Additionally, isotherm modeling was performed and the results indicated the CTS/GO/ZnO composite’s surface is heterogeneous and CO_2_ adsorption occurred as a multi-layer adsorption process. Moreover, the fractional order kinetic model’s most accurate to fitting the experimental CO_2_ adsorption data exhibited the reaction order can’t be an integer number and the adsorption kinetic was influenced by some factors such as heterogeneity and adsorbate-adsorbent interaction. Finally, thermodynamic parameters evaluation such as ΔH°, ΔG°, and ΔS° indicated the exothermic and dominant physisorption mechanism of the CO_2_ uptake on the CTS/GO/ZnO surface. A regeneration study of the CTS/GO/ZnO composite resulted in high stability and robustness of the adsorbent after 10 cycles of adsorption–desorption regarding the minor losses in the CO_2_ adsorption efficiency (around 4.36%).

## Data Availability

Data are available [from Farnoush Fathal] with the permission of [Alireza Hemmati]. The data that support the findings of this study are available from the corresponding author, [Alireza Hemmati and, Hamidreza moghadamzadeh], upon reasonable request.

## List of symbols

A: Temkin model constant (L/mol)
b_T_: Temkin isotherm constant (J/mol)
C_e_: Equilibrium concentration
f: Subscript that refers to final condition
i: Subscript that refers to initial condition
k_L_: Langmuir model constant (bar^−1^)
k_1_: First-order kinetic model rate constant (min^−1^)
k_F_: Freundlich model constant (mmol.g^−1^.bar^−1/n^)
k_2_: Second-order model rate constant (g.mmol^−1^.min^−1^)
k_n_: Fractional-order rate constant [(mmol/g)^(1−n)^ min^−m^]
m: Mass of adsorbed gas(mg)
M_w_: Molecular weight (g/mol)
P: Pressure (bar)
P_e_: Equilibrium pressure (bar)
q: Adsorption capacity (mg/g)
q_e_: Equilibrium adsorption capacity (mmol/g)
q_m_: Maximum adsorption capacity (mmol/g)
q_t_: Adsorption capacity at time t (mmol/g)
R: Gas constant (8.314 J. K^−1^.mol^−1^)
R^2^: Correlation coefficient
T: Temperature (K)
t: Time (second)
w: Mass of adsorbent (g)
Z: Compressibility factor
$$\mathrm{\Delta H}$$: Enthalpy change
$$\mathrm{\Delta S}$$: Entropy change
$\mathrm{\Delta G}$: Gibbs free energy change
α: Elovich model parameter (mmol.g^−1^.min^−1^)
β: Elovich model parameter(g.mmol^-1^)
λ: D − R model constant(mol^2^.J^-2^)
ω: Polanyi potential (KJ.mol^-1^)
CTS: Chitosan
GO: Graphene oxide
ZnO: Zinc oxide
MPV: Micropore volume
APD: Average pore diameter
FTIR: Fourier transform infrared
BET: Brunauer emmett teller
SEM: Scanning electron microscopy
XRD: X-ray diffraction analysis
AARE: Absolute average relative error
